# Maintenance hemodialysis patients’ nutritional literacy and its relationship with frailty: a cross-sectional study based on latent profile analysis

**DOI:** 10.3389/fnut.2026.1807070

**Published:** 2026-05-13

**Authors:** Jiquan Zhang, Wei Qing, Fan Xu

**Affiliations:** 1 Nephrology Department, Deyang People's Hospital, Deyang, Sichuan, China; 2 Oncology Department, Deyang People's Hospital, Deyang, Sichuan, China

**Keywords:** end-stage renal disease, frailty, latent profile analysis, maintenance hemodialysis patients, nutritional literacy

## Abstract

**Background:**

Malnutrition is a common complication among maintenance hemodialysis (MHD) patients. Although the association between nutritional literacy and frailty is well established, prior research has largely focused on individual risk factors or relied solely on total scores from nutrition-related scales, thereby overlooking heterogeneity within patient groups. As a result, the specific relationship between distinct patterns of nutritional literacy and frailty remains unclear.

**Objective:**

To identify distinct nutritional literacy profiles among MHD patients, examine sociodemographic differences across these profiles, and assess their association with frailty.

**Method:**

Between May and September 2024, we recruited a convenience sample of 335 MHD patients from the Department of Nephrology at a tertiary Grade A hospital in Deyang, Sichuan Province. Participants completed questionnaires on demographic characteristics, frailty (assessed using the Tilburg Frailty Indicator [TFI]), and nutritional literacy (measured by the Nutrition Literacy Assessment Questionnaire for Maintenance Hemodialysis Patients [NLAQ-MHD]). Latent profile analysis was used to identify distinct nutritional literacy profiles. Bivariate and multivariable logistic regression models examined associations between profile membership and sociodemographic variables. Differences in frailty across profiles were evaluated using analysis of variance.

**Results:**

Latent profile analysis revealed three distinct nutritional literacy profiles among MHD patients: low nutritional literacy (26.9%), moderate nutritional literacy (59.4%), and high nutritional literacy (13.7%). Multivariable logistic regression showed that education level and Per capita monthly household income were significant predictors of profile membership (*p* < 0.05). Frailty levels differed significantly across the three nutritional literacy profiles.

**Conclusion:**

This study offers new insights into the heterogeneity of nutritional literacy among MHD patients and demonstrates that lower nutritional literacy is associated with greater frailty. These findings not only advance knowledge for future research but also provide a basis for developing targeted frailty intervention strategies in this population.

## 1 Introduction

Population aging and the rising prevalence of chronic conditions such as diabetes and hypertension have led to a substantial increase in the number of maintenance hemodialysis (MHD) patients. According to data from the China National Research Data Service Platform (CNRDS), the hemodialysis prevalence in China reached 519.3 per million population in 2021, with approximately 750,000 patients receiving MHD by the end of that year (1). Annual mortality among hemodialysis patients ranges from 15 to 20% (2), and complications such as malnutrition, infection, and anemia further heighten this risk (3, 4).

Nutrient losses during dialysis, including amino acids, proteins, and vitamins, combined with increased protein catabolism and elevated levels of pro-inflammatory cytokines such as IL-6 (5–8), contribute to a malnutrition prevalence ranging from 14.8 to 70.7% among hemodialysis patients (9–11). At the same time, MHD patients frequently experience various oral health problems, including dry mouth, taste disturbances, and oral infections (12). These local oral conditions are closely associated with overall health status (13) and significantly impair eating experiences and food choices, thereby exacerbating the risk of malnutrition. Although malnutrition markedly increases the risk of cardiovascular events and mortality, it is potentially reversible through early intervention (14, 15). Nevertheless, despite growing awareness of the critical role of nutrition in the prognosis and recovery of dialysis patients, current research and clinical practice have largely emphasized immediate intradialytic nutritional interventions, such as adjustments to dialysate composition and post-dialysis nutritional supplementation, while neglecting sustained assessment and long-term management of nutritional status in MHD patients.

Nutritional literacy refers to an individual’s capacity to obtain, understand, and apply nutrition-related information to maintain and promote health (16). Evidence suggests that nutritional literacy is a key determinant of dietary patterns and behaviors (17). Higher nutritional literacy enables patients to develop scientifically sound dietary plans and ensure adequate nutrient intake, thereby supporting physical recovery and improving quality of life (18). Importantly, MHD patients differ substantially from the general population in terms of nutritional requirements, absorption, and metabolism, and their nutritional status exerts a more pronounced influence on health outcomes. Consequently, early identification and management of nutritional issues in this population hold significant clinical value for enhancing quality of life and reducing the economic burden of disease.

Frailty is defined as an age-related, multidimensional clinical syndrome characterized by a decline in physiological reserve capacity and multisystem dysfunction, which impairs the ability to maintain homeostasis and increases vulnerability to stressors (19). Evidence shows that frailty significantly raises the risk of adverse health outcomes, including delirium, falls, disability, and mortality, and profoundly diminishes patients’ quality of life and life expectancy. It also imposes substantial economic burdens on families and markedly increases healthcare utilization and societal costs (20–22). Among MHD patients, the global prevalence of frailty ranges from approximately 36.5 to 39.6%, and it serves as an independent predictor of poor clinical outcomes (23–25).

Although nutritional status is a key modifiable factor in the onset and progression of frailty among MHD patients (26), and nutritional problems such as nutrient deficiencies, poor dietary habits, unbalanced meals, and excessive alcohol consumption heighten frailty risk (27, 28), traditional research has largely focused on nutritional supplementation itself, paying little attention to the cognitive and behavioral determinants of dietary decision-making. However, nutritional literacy acts as a critical link between knowledge and behavior (29). Studies indicate that higher nutritional literacy supports evidence-based health decisions and improves dietary quality by strengthening nutrition knowledge, critical appraisal skills, and self-efficacy for healthy eating (30, 31). Prior research has demonstrated a significant association between nutritional literacy and frailty. For example, a survey by Duan et al. in older adults revealed an inverse relationship between nutritional literacy and frailty risk (32). Therefore, we hypothesize that nutritional literacy influences frailty status in MHD patients.

Most existing studies have either examined isolated risk factors (such as analysing the impact of socio-demographic data, psychological status and social support on nutritional literacy (33, 34)) or relied on total scores from nutrition literacy scales to assess MHD patients (such as the total score on the nutritional literacy assessment scale for end-stage renal disease dialysis patients (18) and the total score on the dialysis-specific nutrition literacy scale (35)), thereby failing to account adequately for population heterogeneity or the distinct pathways through which different literacy profiles may relate to frailty. Latent Profile Analysis (LPA) is a person-centered statistical method that identifies homogeneous subgroups based on shared underlying characteristics, thereby uncovering patterns of within-population variability (36). This study uses LPA to identify latent nutritional literacy profiles among MHD patients and investigate their associations with frailty. The findings will provide a scientific basis for enhancing nutritional literacy and mitigating frailty in this vulnerable population, while guiding the development of tailored nutritional interventions.

## 2 Method

### 2.1 Participants

This study utilized a cross-sectional design. Between May and September 2024, MHD patients were recruited from the Department of Nephrology at Deyang People’s Hospital in Sichuan Province, China. Inclusion criteria were: (1) meeting the end-stage renal disease (ESRD) diagnostic criteria as outlined in the “Guidelines for Screening, Diagnosis, and Prevention of Chronic Kidney Disease” (37); (2) being aged 18 years or older; (3) undergoing regular dialysis for at least 3 months with a frequency of two or more sessions per week; (4) being mentally competent without psychiatric disorders and possessing basic communication and comprehension abilities; and (5) providing informed consent and voluntarily participating. Exclusion criteria included: (1) physical dysfunction; (2) comorbidities such as other traumatic conditions, tumors, or severe organ failure. The study received approval from the Ethics Committee of Deyang People’s Hospital (Ethics Approval Number: 2022-04-037K01).

**Figure 1:**
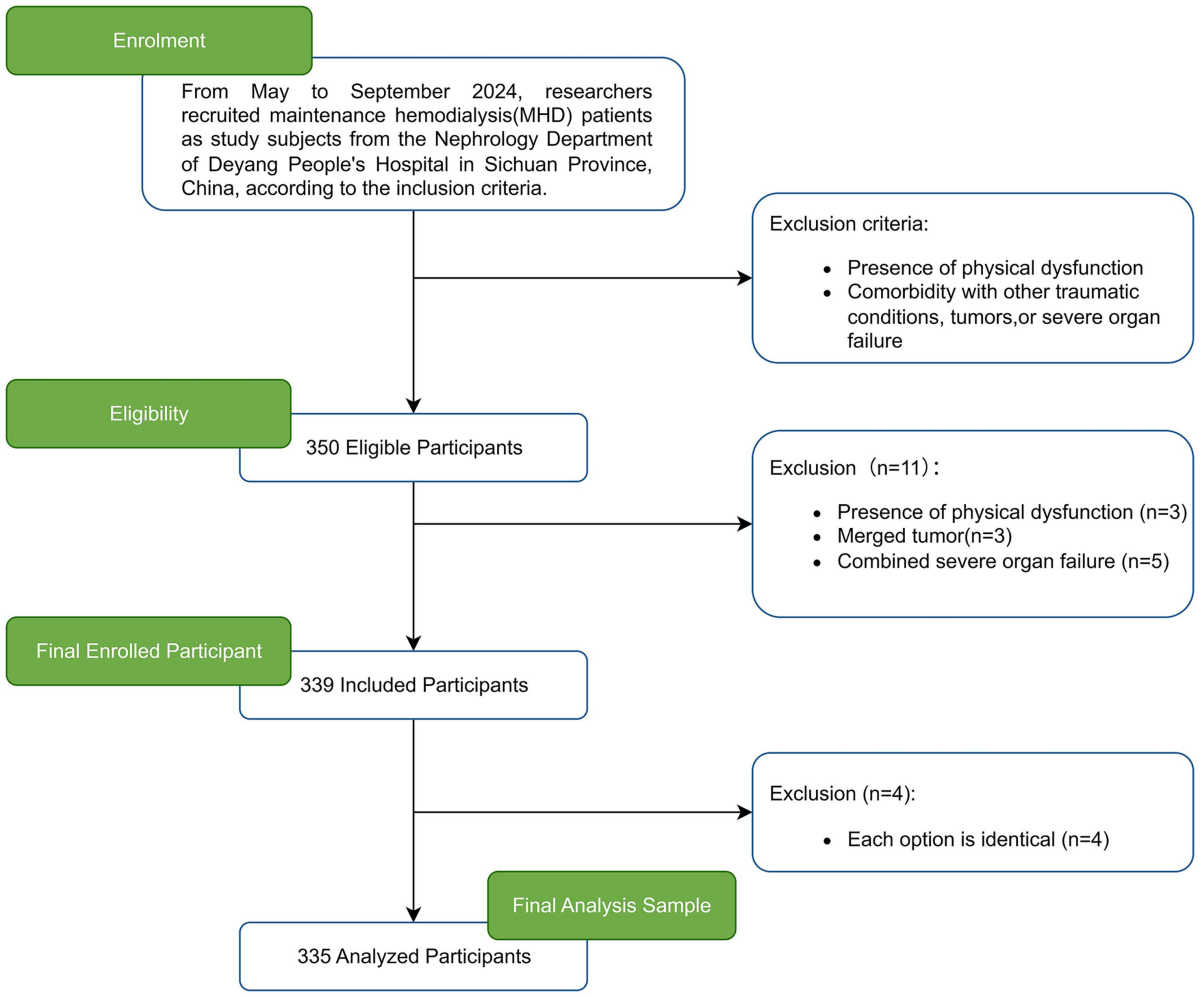
The process of participant selection.

The required sample size was calculated using GPower software (version 3.1) (38), based on a linear regression model. According to Cohen’s standards (39), the test power was set to 0.95 with an alpha value of 0.05 and a medium effect size *f*^2^ of 0.15. Given that this study involved 15 independent variables, GPower determined the necessary sample size to be 199 cases. To account for a 10% attrition rate, the minimum required sample was adjusted to 219. A total of 350 MHD patients were screened for participation. Three patients were excluded due to physical functional impairments (severe cerebrovascular disease sequelae, advanced Parkinson’s disease), three due to concurrent tumors, and five due to severe organ failure (acute liver failure, New York Heart Association [NYHA] Class IV heart failure, severe COPD). After collecting the questionnaires, four participants were excluded for providing identical responses across all items. The final sample comprised 335 valid questionnaires, resulting in a response rate of 95.7%. Figure 1 illustrates the participant screening process.

### 2.2 Measures

#### 2.2.1 General information

This section includes demographic and disease-related data. Demographic data encompass gender, age, education level, marital status, number of children, employment status, per capita monthly household income, religious affiliation, place of residence, living arrangement, length of dialysis, BMI, and number of comorbid chronic diseases.

#### 2.2.2 Tilburg Frailty Indicator (TFI)

TFI is a self-administered frailty assessment tool developed by Gobbens et al. at Tilburg University, the Netherlands, in 2010 (40). The scale consists of 15 items across three dimensions: physical, psychological, and social frailty. Each item is scored dichotomously (0 or 1), yielding a total score ranging from 0 to 15. A score of 5 or higher indicates frailty, with higher scores reflecting greater severity. The original version reported a Cronbach’s alpha coefficient of 0.74. The Chinese version, translated by Xi (41), demonstrated a Cronbach’s alpha of 0.686. Wang et al. administered this Chinese version to MHD patients and obtained a Cronbach’s alpha coefficient of 0.75, indicating acceptable reliability in this population and supporting its suitability for assessing frailty among MHD patients (42). In the present study, the scale yielded a Cronbach’s alpha of 0.683.

#### 2.2.3 MHD patients nutrition literacy assessment questionnaire(NLAQ-MHD)

NLAQ-MHD, developed by Mingyu (43), contains 25 items organized into five dimensions: nutritional attitude, nutritional knowledge, nutritional skills, information interaction, and information evaluation. Responses are rated on a 5-point Likert scale (strongly agree = 5, strongly disagree = 1), with higher total scores indicating better nutritional literacy. The questionnaire demonstrates strong psychometric properties, with a Cronbach’s alpha coefficient of 0.950, split-half reliability of 0.853, test–retest reliability of 0.929, and a content validity index of 0.845.

### 2.3 Data collection

To ensure data quality, researchers provided standardized training to all participating nurses on the inclusion and exclusion criteria, study protocols, and questionnaire administration procedures. During data collection, researchers used standardized instructions, obtained informed consent from participants, and administered questionnaires in person. Any missing or ambiguous responses were clarified with participants immediately to ensure data accuracy and completeness. After collection, two researchers independently verified and entered the data into Excel, and a third researcher performed a final accuracy check.

### 2.4 Data analyses

Data analysis was performed using SPSS version 20.0 and Mplus version 8.0, with a significance level set at *α* = 0.05. Two-tailed tests were used, and results with *p* < 0.05 were considered statistically significant. A latent profile model was constructed in Mplus 8.0. Parameter estimation employed the maximum likelihood (ML) method, with convergence defined as an absolute change in the log-likelihood function between consecutive iterations of less than 0.0001 and a maximum of 1,000 iterations. To avoid local optima, the model used 200 random starting values and 50 final-stage optimizations; all models converged under these settings. The six dimension scores from the NLAQ-MHD served as manifest variables to identify latent nutrition literacy profiles among MHD patients. The analysis began by assuming a single profile. The number of profiles was then increased, and model parameters were evaluated at each step. The optimal number of profiles was selected based on comparative model fit indices. Fit indices for latent profile analysis included the Akaike Information Criterion (AIC), Bayesian Information Criterion (BIC), adjusted Bayesian Information Criterion (aBIC), classification entropy, the Lo–Mendell–Rubin likelihood ratio test (LMR), and the Bootstrap Likelihood Ratio Test (BLRT). Lower values of AIC, BIC, and aBIC indicate better model fit. Entropy ranges from 0 to 1, with values closer to 1 reflecting higher classification accuracy. The LMR and BLRT compare the fit of a *k*-profile model against a (*k* − 1)-profile model (36). When both tests yield *p* < 0.05, the *k*-profile model provides a significantly better fit than the (*k* − 1)-profile model. Statistical analyses were conducted using SPSS 20.0. Continuous variables are presented as mean ± standard deviation, and categorical variables as frequency and percentage. Between-group comparisons of sociodemographic characteristics across latent profiles were performed using chi-square tests. Multivariable logistic regression was then conducted, with latent profile membership as the dependent variable and sociodemographic factors showing significant differences in the chi-square tests as independent variables, to identify predictors of nutrition literacy profile membership. Additionally, one-way analysis of variance (ANOVA) was used to examine differences in frailty scores across the identified nutrition literacy profiles among MHD patients.

## 3 Results

### 3.1 Basic information

The study included 335 MHD patients, of whom 204 (60.9%) were male and 131 (39.1%) were female. Ages ranged from 18 to 93 years (mean = 59.63 ± 12.33). A total of 43.3% had completed elementary school or less. Most participants were married (80.9%), and 67.2% had one child. Nearly half (42.1%) were farmers, and 54.0% reported a per capita monthly household income below 2,000 yuan. The majority (90.4%) reported no religious affiliation. In terms of residence, 37.9% lived in urban areas, 28.1% in towns, and 34.0% in rural areas. Most patients (73.7%) lived with their spouse and/or children. Slightly more than half (52.5%) had a BMI in the normal range, about half (50.1%) had been receiving dialysis for more than 60 months, and 44.5% had two comorbid chronic diseases. Details are presented in Table 1.

**Table 1:** Demographic characteristics of the participants (*n* = 335).

Variables	Categories	Total samples (*n* = 335)	Profile 1 (*n* = 90)	Profile 2 (*n* = 46)	Profile 3 (*n* = 199)	*χ*^2^	*p*
		*n* (%)	*n* (%)	*n* (%)	*n* (%)		
Gender	Male	204 (60.9)	54 (60.0)	30 (65.2)	120 (60.3)	0.421	0.810
	Female	131 (39.1)	36 (40.0)	16 (34.8)	79 (39.7)		
Age (years)	18–59	166 (49.6)	44 (48.9)	26 (56.6)	96 (48.2)	1.047	0.593
	≥60	169 (50.4)	46 (51.1)	20 (43.5)	103 (51.8)		
Education level	Elementary school or below	145 (43.3)	49 (54.4)	10 (21.7)	86 (43.2)	40.249	<0.001
	Middle school	133 (39.7)	34 (37.8)	14 (30.4)	85 (42.7)		
	College or above	57 (17.0)	7 (7.8)	22 (47.8)	28 (14.1)		
Marital status	Married	271 (80.9)	70 (77.8)	38 (82.6)	163 (81.9)	0.786	0.675
	Divorced/Widowed/ Unmarried	64 (19.1)	20 (22.2)	8 (17.4)	36 (18.1)		
Number of children	0	4 (1.2)	2 (2.2)	1 (2.2)	1 (0.5)	4.456^*^	0.615
	1	225 (67.2)	57 (63.3)	34 (73.9)	134 (67.3)		
	2	81 (24.2)	25 (27.8)	9 (19.6)	47 (23.6)		
	≥3	25 (7.5)	6 (6.7)	2 (4.3)	17 (8.5)		
Employment status	Farmer	141 (42.1)	40 (44.4)	22 (47.8)	79 (39.7)	2.778	0.836
	Employed	44 (13.1)	14 (15.6)	6 (13.0)	24 (12.1)		
	Unemployed	64 (19.1)	16 (17.8)	7 (15.2)	41 (20.6)		
	Retirement	86 (25.7)	20 (22.2)	11 (23.9)	55 (27.6)		
Per capita monthly household income (yuan)	<2,000	181 (54.0)	52 (57.8)	14 (30.4)	115 (57.8)	21.419	<0.001
	2,000–5,000	101 (30.1)	29 (32.2)	15 (32.6)	57 (28.6)		
	>5,000	53 (15.8)	9 (10.0)	17 (37.0)	27 (13.6)		
Religious affiliation	No	303 (90.4)	84 (93.3)	39 (84.8)	180 (90.5)	2.576	0.276
	Yes	32 (9.6)	6 (6.7)	7 (15.2)	19 (9.5)		
Place of residence	Urban	127 (37.9)	33 (36.7)	13 (28.3)	81 (40.7)	3.347	0.502
	Town	94 (28.1)	23 (25.6)	16 (34.8)	55 (27.6)		
	Rural	114 (34.0)	34 (37.8)	17 (37.0)	63 (31.7)		
Living arrangement	Living with spouse	124 (37.0)	33 (36.7)	17 (37.0)	74 (37.2)	3.912	0.865
	Living with children	37 (11.0)	12 (13.3)	5 (10.9)	20 (10.1)		
	Living with spouse and children	86 (25.7)	18 (20.0)	12 (26.1)	56 (28.1)		
	Live alone	54 (16.1)	18 (20.0)	6 (13.0)	30 (15.1)		
	Others	34 (10.1)	9 (10.0)	6 (13.0)	19 (9.5)		
length of dialysis (month)	<36	93 (27.8)	33 (36.7)	7 (15.2)	53 (26.6)	8.519	0.074
	36–60	74 (22.1)	19 (21.1)	14 (30.4)	41 (20.6)		
	>60	168 (50.1)	38 (42.2)	25 (54.3)	105 (52.8)		
BMI	Underweight	25 (7.5)	7 (7.8)	5 (10.9)	13 (6.5)	2.692	0.846
	Normal	176 (52.5)	46 (51.1)	20 (43.5)	110 (55.3)		
	Overweight	99 (29.6)	27 (30.0)	15 (32.6)	57 (28.6)		
	Obesity	35 (10.4)	10 (11.1)	6 (13.0)	19 (9.5)		
Number of comorbid chronic diseases	1	63 (18.8)	10 (11.1)	13 (28.3)	40 (20.1)	8.107	0.088
	2	149 (44.5)	48 (53.3)	19 (41.3)	82 (41.2)		
	≥3	123 (36.7)	32 (35.6)	14 (30.4)	77 (38.7)		

^*^Indicates corrected *χ*^2^ test.

### 3.2 Latent profiling and naming of MHD patients’ nutritional literacy

Latent profile models with one to five categories were estimated using the five dimension scores from the NLAQ-MHD as manifest variables. Results are shown in Table 2. As the number of latent categories increased from one to five, the AIC, BIC, and aBIC values steadily decreased. The three-category model yielded the highest entropy (0.797). Moreover, both the LMR and BLRT reached statistical significance for the two- and three-category models, but the LMR *p* value exceeded 0.05 for the four-category model. Based on these fit indices, the three-category model was selected as the optimal solution. See Table 2.

**Table 2:** Latent profiling of MHD patients’ nutritional literacy fitting indices.

Model	Loglikelihood	AIC	BIC	aBIC	Entropy	LMR	BLRT	Profile probability
1-profile	−4,073.384	8,166.769	8,204.91	8,173.189	–	–	–	
2-profile	−3,894.266	7,820.532	7,881.558	7,830.804	0.758	0.000	0.000	0.421\0.579
**3-profile**	**−3,839.95**	**7,723.9**	**7,807.811**	**7,738.025**	**0.797**	**0.007**	**0.000**	**0.269\0.137\0.594**
4-profile	−3,824.825	7,705.65	7,812.445	7,723.627	0.701	0.135	0.000	0.206\0.388\0.304\0.102
5-profile	−3,815.86	7,699.72	7,829.401	7,721.549	0.718	0.627	0.167	0.221\0.051\0.373\0.263\0.092

The model solution chosen is bolded.

**Figure 2:**
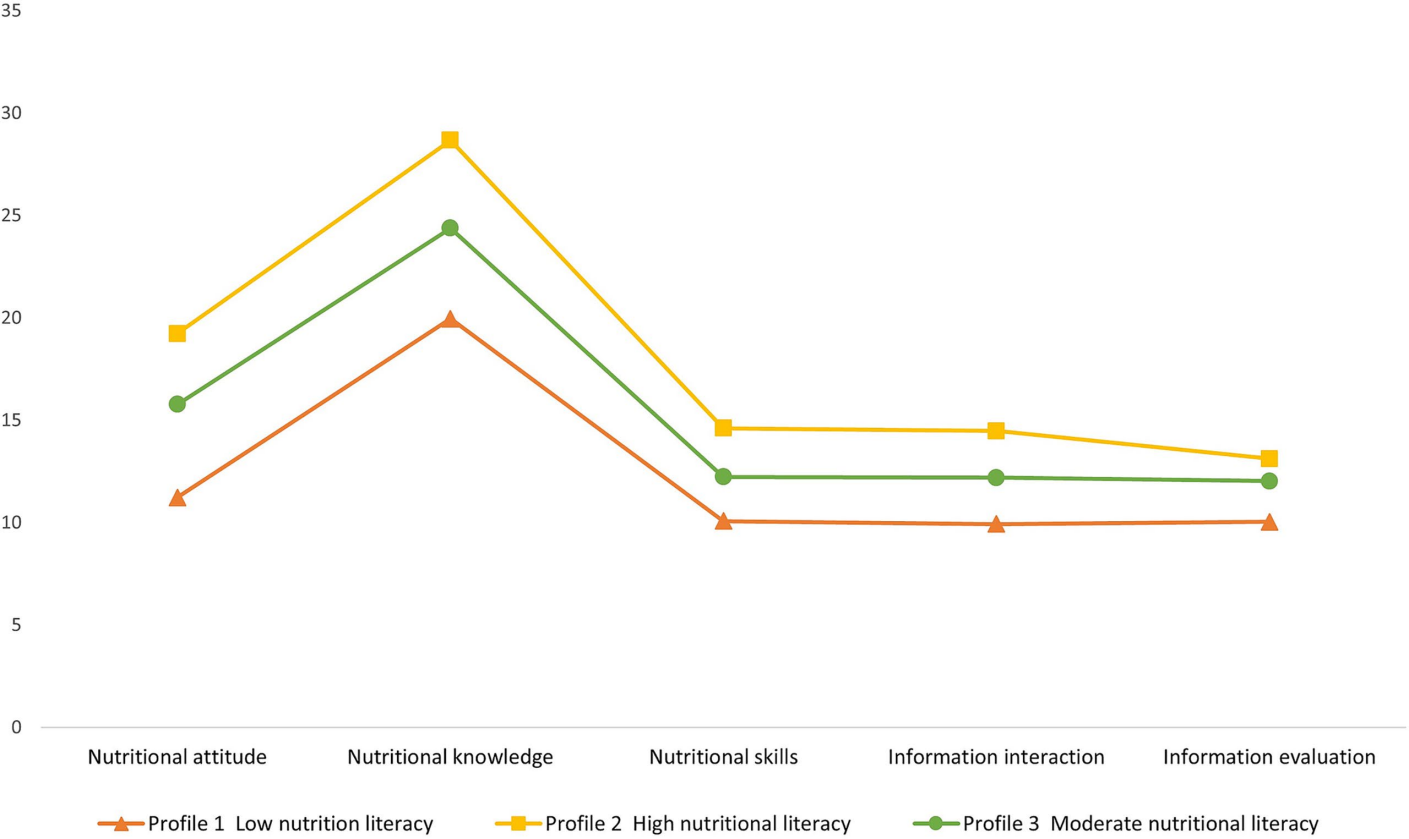
Three potential categories of nutrition literacy among MHD patients.

The three latent categories were named based on their score patterns across the NLAQ-MHD dimensions. Category 1 (*n* = 90, 26.9%) showed low scores on all dimensions and was labeled “Low nutrition literacy.” Category 2 (*n* = 46, 13.7%) displayed consistently high scores across all dimensions and was designated “High nutrition literacy.” Category 3 (*n* = 199, 59.4%) exhibited moderate scores across all dimensions and was named “Moderate nutrition literacy.” See Figure 2.

### 3.3 Bivariate analysis of different latent profiles of MHD patients’ nutritional literacy

The three MHD patient groups differed significantly in education level and per capita monthly household income (*p* < 0.05). See Table 1.

### 3.4 Multivariate logistic regression analysis of latent profiles of MHD patients’ nutritional literacy

To assess potential multicollinearity, collinearity diagnostics were performed for all independent variables before regression analysis. The variance inflation factor (VIF) was below 5 for every variable, indicating no serious multicollinearity (44). Multinomial logistic regression was conducted to identify factors associated with nutrition literacy profiles, with nutrition literacy category as the dependent variable (low = 1, high = 2, moderate = 3; low nutrition literacy as the reference group). Variables that showed statistical significance in bivariate analyses were entered into the multivariate model. Results indicated that patients with elementary school education or lower (OR = 0.091, *p* < 0.001) and those with middle school education (OR = 0.160, *p* < 0.05) were significantly more likely to fall into the low nutrition literacy group compared to patients with college education or higher. Likewise, patients with a per capita monthly household income below 2,000 yuan (OR = 0.259, *p* < 0.05) were more likely to belong to the low nutrition literacy group than those with an income above 5,000 yuan. Detailed results are presented in Table 3.

**Table 3:** Multiple logistic regression analysis of latent profiles of MHD patients’ nutritional literacy.

Variables	Profile 1 and 2				Profile 1 and 3			
	*β*	OR	95%CI	*p*	*β*	OR	95%CI	*p*
Education level (Reference: college or above)								
Elementary school or below	−2.395	0.091	(0.030, 0.281)	<0.001	−0.826	0.438	(0.175, 1.097)	0.078
Middle school	−1.833	0.160	(0.055, 0.468)	0.001	−0.464	0.629	(0.249, 1.588)	0.326
Per capita monthly household income (yuan) (Reference: >5,000)								
<2,000	−1.351	0.259	(0.089, 0.754)	0.013	−0.135	0.874	(0.376, 2.030)	0.754
2,000–5,000	−0.998	0.369	(0.125, 1.083)	0.070	−0.34	0.712	(0.294, 1.727)	0.452

Profile 1:“Low nutrition literacy”, Profile 2:“High nutrition literacy”, Profile 3:“Moderate nutrition literacy”.

### 3.5 Relationships between latent profiles of MHD patients’ nutritional literacy and frailty

MHD patients with different nutrition literacy profiles showed statistically significant differences in total frailty scores as well as in the physical and social frailty subscale scores (*p* < 0.05). See Table 4.

**Table 4:** Differential analysis of three patterns of nutritional literacy and frailty.

Variable	Profile 1	Profile 2	Profile 3	*F*	*p*	Pairwise comparison
Physical frailty	2.91 ± 2.18	1.76 ± 1.70	1.94 ± 1.88	8.995	<0.001	1 > 2,3
Psychological frailty	1.30 ± 1.09	0.96 ± 0.94	1.22 ± 0.98	1.846	0.160	–
Social frailty	0.98 ± 0.85	0.57 ± 0.72	0.82 ± 0.75	4.355	0.014	1 > 2,3
Total score	5.19 ± 2.91	3.28 ± 2.56	3.98 ± 2.79	8.689	<0.001	1 > 2,3

Profile 1:“Low nutrition literacy”, Profile 2: “High nutrition literacy”, Profile 3:“Moderate nutrition literacy”.

## 4 Discussion

MHD patients face substantial challenges across medical, financial, social, and psychological domains, and often experience significant nutritional difficulties. Evidence suggests that the prevalence of malnutrition among MHD patients ranges from 14.8 to 70.7% (11–13). Nutrition literacy, a higher-order determinant of nutritional status, plays a critical role in strengthening patients’ self-management capacity, improving nutritional intake, and optimizing clinical outcomes (45). Enhancing nutrition literacy in this population is therefore essential for reducing malnutrition rates and mitigate dialysis-related complications (46). Using LPA, this study identifies distinct subtypes of nutrition literacy among MHD patients and examines their associations with frailty status and related factors. The findings aim to inform the development of tailored nutritional interventions, ultimately contributing to lower malnutrition incidence and fewer dialysis complications.

In this study, nutrition literacy among MHD patients was classified into three latent profiles: “low nutrition literacy” (26.9%), “moderate nutrition literacy” (59.4%), and “high nutrition literacy” (13.7%), highlighting substantial heterogeneity within the population. The moderate nutrition literacy group constituted the largest subgroup (59.4%), a finding consistent with results reported by Xia et al. (47). These patients demonstrated basic nutritional knowledge and could apply fundamental principles of food selection, yet they struggled to deeply interpret, critically evaluate, or consistently implement nutritional information in daily practice, reflecting an intermediate state characterized by high awareness but incomplete internalization. Evidence suggests that behavioral skills training grounded in the Knowledge–Attitude–Practice (KAP) model and social cognitive theory can effectively support the translation of knowledge into sustained action (48, 49). To promote this transition, healthcare providers should incorporate structured approaches such as dietary diary keeping, hands-on practice using the food exchange method, and simulated decision-making exercises focused on interdialytic weight gain. These strategies allow patients to rehearse real-world choices in clinically relevant contexts. In parallel, setting incremental behavioral goals, for example, managing phosphorus and potassium intake and adhering to fluid restrictions, can help patients build a “knowledge–confidence–behavior” pathway, thereby facilitating the shift from cognitive understanding to habitual practice.

The low nutrition literacy group accounted for 26.9% of the sample and represents a high-risk population for malnutrition. Prior research confirms that many hemodialysis patients in this group have limited mastery of basic nutritional knowledge and practical skills, lack the ability to make scientifically informed food choices, and show inadequate understanding of dietary management guidelines specific to dialysis (50). In health-related information exchanges, these individuals rarely initiate communication with healthcare providers and struggle to critically evaluate nutritional content, often unable to judge the scientific validity or reliability of information sources. According to the Health Literacy Skills Framework, limited communication and interaction skills mediate the link between low health literacy and adverse health outcomes. Patients with low health literacy frequently encounter barriers in clinician–patient communication and participate minimally in shared decision-making (51). As a result, they are more likely to consume foods high in sodium, potassium, and phosphorus, which increases renal metabolic burden and accelerates disease progression. Given their limited foundational knowledge, clinical staff should adopt a “less but more precise” educational approach for this group. Concrete tools, such as food models and dietary pyramid diagrams, can simplify complex nutritional concepts while emphasizing core principles of the dialysis diet and essential cooking techniques, thereby lowering the threshold for applying knowledge in daily life. Additionally, because these patients tend to be passive about nutrition and engage infrequently in seeking health information, healthcare providers should proactively establish a routine follow-up system. Regular phone calls, home visits, or partnerships with community resources can help build trust, gradually correct misconceptions about nutrition, and support sustained improvements in nutrition literacy.

The high nutrition literacy group constituted the smallest subgroup at 13.7%. These individuals achieved the highest scores across all five dimensions, nutritional attitude, knowledge, skills, information interaction, and evaluation, likely due to their higher levels of education and income, which enabled them to actively access and apply nutrition- and health-related knowledge through available social resources. Despite their overall strength, this group showed a relative weakness in information evaluation, suggesting a need for further development in critically appraising nutritional content. According to Nutbeam’s Health Literacy Hierarchy Model, critical health literacy involves the advanced cognitive capacity to analyze, evaluate, and question health information, a key competency that empowers MHD patients to distinguish credible from misleading claims and make evidence-based decisions in an environment saturated with health messaging (52). Accordingly, instruction should continue to support these patients in identifying trustworthy nutritional information, assessing the reliability of sources, and filtering scientifically valid content from the broader information landscape. In addition, a peer education empowerment program could be implemented by inviting members of this group to serve as peer mentors; their lived experience and modeling could provide practical guidance for patients with lower literacy levels while reinforcing and refining their own application of knowledge through teaching.

MHD patients with elementary school education or lower (OR = 0.091, *p* < 0.001) and those with middle school education (OR = 0.160, *p* < 0.05) were significantly more likely to exhibit low nutrition literacy than patients with college-level education or higher. This aligns with findings from comparable studies identifying educational attainment as a key determinant of nutrition literacy (53). Two interrelated mechanisms help explain this association. First, patients with lower educational backgrounds often have reduced capacity to access and process health information, encountering substantial difficulties in understanding abstract nutritional concepts and effectively interpreting nutrition-related content (54), which contributes to lower nutrition literacy. Second, limited formal education is frequently associated with less exposure to critical thinking development and lower self-efficacy, resulting in diminished motivation to engage with nutritional learning and greater challenges in comprehending complex dietary information (55), factors that further hinder the acquisition and improvement of nutrition literacy. Given these barriers, nutrition education for MHD patients with lower educational attainment should prioritize intuitive and actionable strategies. Visual aids such as plate models and cooking demonstration videos can translate abstract nutritional principles into concrete, step-by-step practices to support skill building. For patients with higher education but suboptimal dietary behaviors, interventions should focus on cognitive restructuring through techniques like motivational interviewing to help them recognize and resolve discrepancies between their knowledge and actual eating habits.

MHD patients with a per capita monthly household income below 2,000 yuan (OR = 0.259, *p* < 0.05) were more likely to exhibit low nutrition literacy than those with an income exceeding 5,000 yuan. This finding is consistent with prior research linking household income to nutrition literacy (56, 57). The association may arise because limited financial resources constrain patients’ ability to access high-quality nutritious foods and reliable nutritional information. Evidence suggests that MHD patients often experience substantial health-related financial toxicity; individuals in low-income groups tend to prioritize essential medical expenditures, which severely limits the resources available for acquiring nutritional knowledge, purchasing healthy foods, or engaging in nutrition education programs (58), ultimately contributing to lower nutrition literacy. Addressing this disparity requires strengthening both economic security and access to nutritional resources. Policymakers could reduce the cost burden of healthy eating through preferential insurance coverage or targeted nutritional subsidies. At the same time, fostering partnerships between healthcare systems and community organizations to deliver low-cost, community-based nutrition education may enhance patients’ awareness and prioritization of dietary self-management. Additionally, harnessing social media and local community networks to create platforms for sharing healthy eating experiences can promote peer support and broaden exposure to positive dietary role models.

Additionally, this study did not identify a statistically significant association between BMI and nutrition literacy categories. A survey of adolescents similarly reported no link between nutrition literacy and BMI (59), although other studies have suggested that nutrition literacy may be associated with obesity or overweight in adolescent populations (60). These inconsistent findings may reflect differences in study populations, geographic settings, and assessment instruments. Moreover, BMI is not an optimal indicator for assessing nutritional status in patients undergoing maintenance hemodialysis. Because BMI is derived solely from height and weight, it cannot differentiate between adipose tissue and lean body mass, particularly muscle mass. In dialysis patients, body weight is frequently affected by fluid retention and shifts in hydration status, whereas loss of muscle mass more accurately signals malnutrition risk. As a result, reliance on BMI alone may mask patients’ true nutritional intake and metabolic condition. Future research should therefore incorporate more precise measures of body composition, such as bioelectrical impedance analysis (BIA), or direct assessments of muscle mass to better elucidate how nutritional status influences the actual nutritional condition and clinical outcomes of dialysis patients.

This study found significant differences in frailty levels among MHD patients across nutrition literacy profiles. *Post hoc* analyses showed that patients with low nutrition literacy had markedly higher frailty scores than those with moderate or high nutrition literacy, indicating that nutrition literacy is a key determinant of frailty in this population, a finding consistent with Xia’s research (47). Unlike frailty in individuals with general chronic conditions, which often reflects age-related physiological decline, frailty in MHD patients arises from the interplay between ESRD-specific factors and nutritional status (61). The prevalence of frailty among MHD patients ranges from 36.5 to 39.6% (23, 24) and is strongly linked to malnutrition, protein-energy wasting (PEW), and cognitive impairment (62, 63). Managing the complex dietary regimen required for hemodialysis, balancing electrolyte restrictions while ensuring adequate protein and energy intake, demands a high level of nutrition literacy to understand and implement these requirements accurately. Poor adherence to dietary recommendations commonly leads to PEW, which can precipitate sarcopenia (64), an established independent risk factor for frailty (65). Yet only 34.9% of MHD patients achieve good dietary compliance, and nutrition literacy influences dietary behavior indirectly through self-efficacy and self-management skills (35). Patients with low nutrition literacy often lack the ability to accurately interpret food nutrient content and struggle to follow prescribed dietary limits, making them vulnerable to nutritional imbalances such as inadequate protein intake and electrolyte disturbances, thereby elevating frailty risk. In contrast, those with high nutrition literacy are better equipped to plan meals, select appropriate foods and portion sizes, and maintain overall nutritional balance. Importantly, nutrition literacy in MHD patients demonstrates a unique form of dynamic vulnerability: as dialysis duration increases, prolonged exposure to strict dietary restrictions may lead to decision fatigue, reducing patients’ capacity to comprehend and act on complex nutritional guidance (66, 67). At the same time, recurrent microinflammatory states, metabolic acidosis, and the accumulation of uremic toxins during dialysis progressively deplete physiological reserves (68, 69). Accordingly, nutritional interventions for MHD patients should extend beyond knowledge transfer and prioritize strengthening self-efficacy alongside structured self-management training to support precise nutritional balance within the constraints of a complex diet, ultimately helping to slow the progression of frailty.

## 5 Conclusion

This study examined heterogeneity in nutrition literacy among MHD patients and its association with frailty. Latent profile analysis identified three distinct nutrition literacy profiles: “low nutrition literacy” (26.9%), “moderate nutrition literacy” (59.4%), and “high nutrition literacy” (13.7%), revealing considerable variation across the patient population. Education level and average monthly household income emerged as key determinants of these profiles, which in turn were significantly associated with frailty severity. The results support the need for healthcare providers to tailor nutritional interventions to the specific characteristics of each group, integrating routine nutrition literacy screening, delivering targeted education, and strengthening patients’ capacity to manage their dietary needs, to help slow the progression of frailty. Future research should further investigate mediating and moderating factors in the relationship between nutrition literacy and frailty among MHD patients, including social support, self-efficacy, and psychological resilience. Such work would deepen understanding of the underlying mechanisms and guide the development of more precise, personalized intervention strategies.

## 6 Limitations

This study has several limitations. The research was constrained by available resources and time, resulting in a single-center cross-sectional design with participants recruited exclusively from the nephrology department of one tertiary hospital, which may limit the generalizability of the findings. Future prospective cohort studies involving multiple centers and larger, more diverse samples are needed to assess the stability of the association between latent nutrition literacy profiles and frailty across different populations. Because the study used a cross-sectional approach, it cannot establish temporal precedence or support causal inferences between nutrition literacy and frailty, nor does it capture how nutrition literacy profiles may evolve throughout the dialysis trajectory; longitudinal follow-up is therefore necessary to examine the dynamic interplay between these variables. Data collection relied primarily on self-reported questionnaires, which are susceptible to recall bias and social desirability bias. Moreover, the Cronbach’s alpha for the TFI scale was close to the conventional lower threshold for acceptable internal consistency, potentially leading to a slightly conservative estimate of frailty levels. To address these measurement concerns, future work should integrate subjective reports with objective assessments, such as Kt/V, mid-upper arm muscle circumference, grip strength, and body composition analysis, to provide a more comprehensive evaluation. Lastly, although this study identified distinct latent profiles of nutrition literacy, it did not investigate whether demographic factors moderate or mediate the relationship between nutrition literacy and frailty. Subsequent research could apply structural equation modeling to systematically clarify the underlying mechanisms linking nutrition literacy to frailty, thereby informing the development of precision-targeted interventions.

## Data Availability

The raw data supporting the conclusions of this article will be made available by the authors, without undue reservation.
